# Immune thrombocytopenia associated with Hashimoto thyroiditis in a pediatric patient: A case report

**DOI:** 10.1097/MD.0000000000026140

**Published:** 2021-06-04

**Authors:** Zhiqing Tian, Hu Gao, Dongqiong Xiao, Xihong Li

**Affiliations:** Department of Emergency/Key Laboratory of Birth Defects and Related Diseases of Women and Children (Ministry of Education), West China Second University Hospital, Sichuan University, Chengdu, China.

**Keywords:** children, Hashimoto thyroiditis, immune thrombocytopenia

## Abstract

**Rationale:**

Immune thrombocytopenia (ITP) is one of the most commonly acquired bleeding diseases in children. Infection and autoimmune disorders are the most common causes of ITP. The pathogenic mechanism of ITP is complex and is not completely understood. Understanding the underlying causes or disorders of ITP will improve the prognosis and make therapy more targeted.

**Patient concerns:**

An 8-year-old girl with ITP responded poorly to first- and second-line treatment. The patient showed multiple scattered petechiae, ecchymoses, and purpura in the skin and blood clots in the oral mucous membrane.

**Diagnoses:**

The patient was diagnosed with ITP associated with Hashimoto thyroiditis.

**Interventions:**

The patient was admitted to our emergency department and received platelet transfusion, IVIG, glucocorticoids and eltrombopag. The patient's thrombocytopenia resolved within 18 days after the administration of levothyroxine treatment.

**Outcomes:**

The patient was diagnosed with Hashimoto thyroiditis, and the platelet count recovered on the 3rd day of levothyroxine treatment. The platelet count became steadily normal with levothyroxine and prednisone treatment within 2 months of follow-up.

**Lessons:**

Early identification of the underlying reasons and treatment with multiple modalities may be useful in improving the prognosis of ITP. The treatment of thyroid disease and restoration of the euthyroid state impact the clinical outcome of ITP in children.

## 1 Introduction

Immune thrombocytopenia (ITP) is a common immune-mediated hemorrhagic disorder in children, occurring in 5% to 10% per 100,000 children annually, and it is primarily characterized by an isolated decrease in the platelet count.^[1,2]^ ITP is more likely to spontaneously recover without intervention in children than adults, and approximately 69% of children with ITP achieve complete remission within 6 months.^[3]^ The management of diagnosed ITP is a “watch-and-wait” careful observation because severe bleeding occurs in only 0% to 4% of children.^[1,3]^ ITP is classified as primary or secondary depending on its association with other diseases or drug exposure, but the treatments are similar for both types.^[4]^ However, if ITP is secondary to an ongoing underlying disease (e.g., infections, autoimmune diseases, or drugs), treatment often focuses on the underlying disease instead of ITP.^[1]^

Infection is the most common causes of secondary ITP in children, and other conditions, such as autoimmune disorders, are rare.^[5]^ Although it is rare, ITP was also associated with thyroid disorders.^[6,7]^ However, subclinical Hashimoto thyroiditis as the cause of secondary ITP is a very rare phenomenon, and no cases of Hashimoto thyroiditis associated with newly diagnosed pediatric ITP were reported. We present a case of an 8-year-old girl who was admitted with severe newly diagnosed ITP and showed a poor response to first- and second-line treatment. This girl was diagnosed with subclinical Hashimoto thyroiditis and was treated with levothyroxine. She showed significantly improved platelet counts over time. This case raises awareness of a rare, but potential, cause for ITP.

## 2 Case report

**Figure 1:**
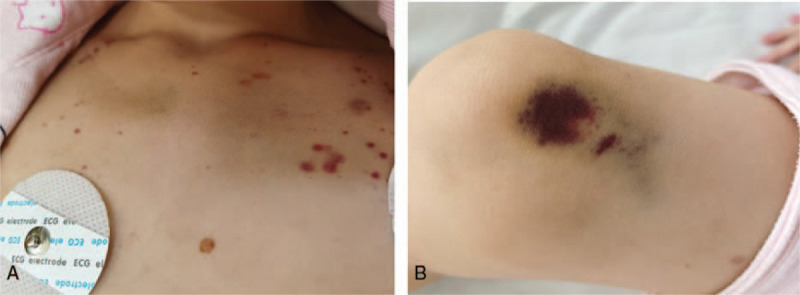
Skin ecchymosis and petechiae appearing on the chest (A) and knee (B) of the patient.

An 8-year-old girl was admitted to the emergency department at our hospital with complications of recurrent petechiae and purpura (Fig. 1). She denied any similar past medical or family history. She was not taking long-term drug therapy and denied receiving any recent vaccinations. She had no recent upper respiratory symptoms or fever. Physical examination revealed multiple scattered petechiae, ecchymoses, and purpura on the skin and blood clots in the oral mucous membrane. Other examination results were unremarkable.

The following laboratory findings at a local hospital were obtained: the complete blood count showed that the platelet count had dropped to 38 × 10^9^/L; anti-*M. pneumoniae* immunoglobulin (Ig)M antibodies were positive; the results of tests for *Legionella pneumophila*, *Rickettsia*, *Chlamydia pneumoniae*, adenovirus, respiratory syncytial virus, influenza A virus, influenza B virus, and parainfluenza virus (type 1,2,3) virus were negative; and clotting function screening was normal. The following laboratory findings were obtained in our hospital: a complete blood count on the day of presentation showed a white blood count of 11.5 × 10^9^/L, hemoglobin of 123 g/L, platelet count of 1 × 10^9^/L, and C-reactive protein of <0.8 mg/L. Liver function, kidney function, blood clotting profile, and blood coagulation factor screening were normal. Tests for hepatitis B and C viruses, HIV, and syphilis yielded negative results.

**Figure 2:**
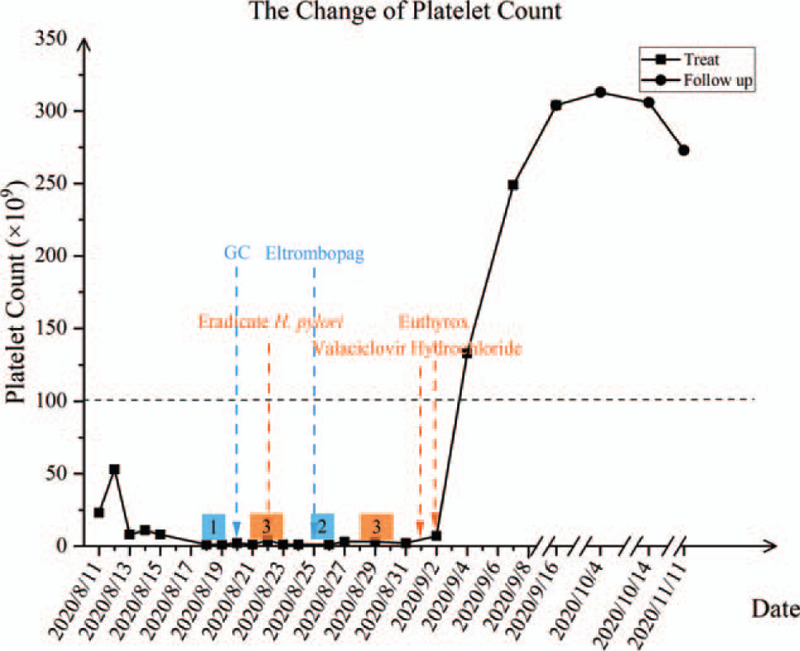
Platelet counts and treatments during emergency department admission. She was treated with platelet transfusion (1), IVIG (2), glucocorticoids (GC), eltrombopag, amoxicillin+clarithromycin+omeprazole (to eradicate H. pylori), valaciclovir hydrochloride, azithromycin (3), and euthyrox at this time.

ITP was considered in this patient based on the clinical manifestations, careful history, physical examination and laboratory findings, especially platelet counts below 100  × 10^9^/L but normal white and red blood cells.^[4]^ The patient received methylprednisolone (dosage unknown) and intravenous Ig (IVIG) (400 mg/kg × 4 days) therapy in the local hospital. However, her platelet count showed a progressive downward trend and decreased to a nadir of 6 × 10^9^/L. The petechiae and purpura worsened. The patient received a platelet transfusion in our hospital after the lack of effect of the IVIG and methylprednisolone treatment in the other hospital and notable mucocutaneous hemorrhage. Unfortunately, the platelet count does not increase. She received 2 units of platelet transfusions in 2 successive 2 days. The platelet count was as low as 2 × 10^9^/L. Pulse methylprednisolone (250 mg) was administered on days 3 and 4, followed by 360 mg on days 5 and 6 and 125 mg on day 7. However, severe thrombocytopenia was unresponsive to these treatments, and the platelet count dropped to 1 × 10^9^/L. The patient received 25 mg eltrombopag daily beginning on day 8, and IVIG (1 g/kg × 2 d) treatment was again considered a possible substandard treatment in another hospital. Corticosteroid maintenance therapy was then added to her treatment. Unfortunately, thrombocytopenia was unresponsive to these treatments, and the platelet count dropped to 1 × 10^9^/L (Fig. 2).

Further laboratory data included the following values: positive antinuclear antibodies (ANAs) with a titer of 1:100, and negative nRNP/Sm, Smith, SS-A (Ro), SS-B (La), Scl-70, Jo-1, Ro-52, CENPB, PCNA, PM-Scl, NU, M2, HI, dsDNA, RIB, and dsDNA. Nasopharyngeal polymerase chain reaction was negative for *M. pneumoniae*, *Chlamydia pneumoniae*, and Epstein-Barr virus. Serologies for influenza A and B, parainfluenza viruses 1, 2, and 3, adenovirus, and respiratory syncytial virus antigen tests were negative. The serological workup for IgM antibody was negative for cytomegalovirus, rubella virus and Epstein-Barr virus. The titer of anti-*M. pneumoniae* IgG antibody was 1:1280 (normal range≤1:160). Platelet-associated Ig was positive. Serum anti-*Helicobacter pylori* (*H. pylori)* testing was positive (1.97, normal range <1.0). Tests of the titer values of serum IgM for herpes simplex virus (HSV) were likely positive (0.98 index, normal<0.9 index). CT scans of the head, chest, and abdomen showed no obvious abnormalities.

Considering the poor treatment response, we added a triple proton pump inhibitor/amoxicillin/clarithromycin therapy for anti-*H. pylori*. Valaciclovir hydrochloride dispersible tablets are effective against HSV infection. The patient received sequential azithromycin for the treatment of *M. pneumoniae* infection (10 mg/kg/day for 3 days, withdrawal for 4 days). However, the platelet counts remained refractory.

**Figure 3:**
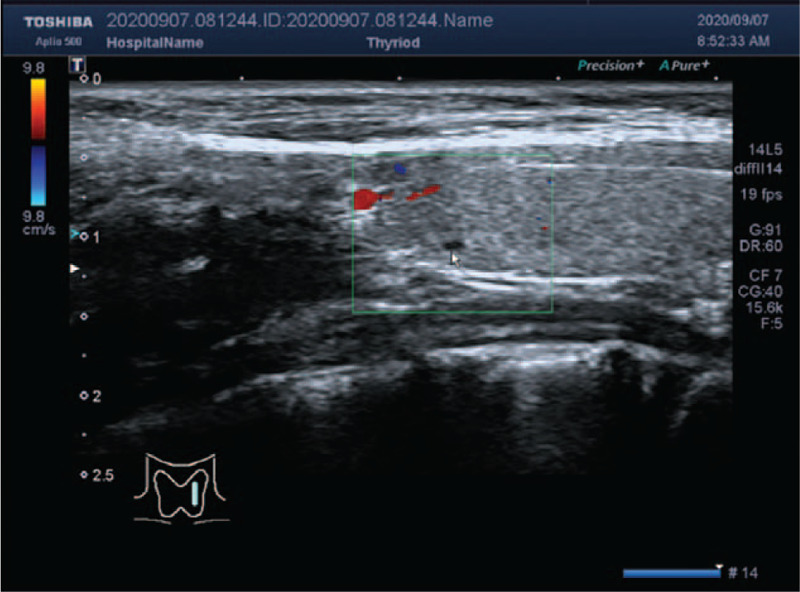
Thyroid gland ultrasound examination in the patient. The white arrow indicates a weak-echo nodule in the left lobe of her thyroid gland.

**Figure 4:**
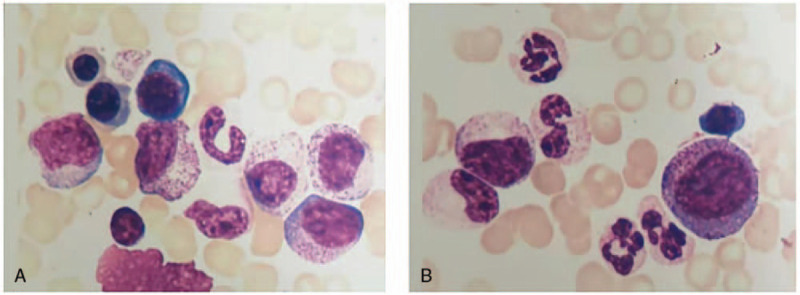
Histological findings of the bone marrow examination in our patient. Magnification: (A, B) ×40, hematoxylin and eosin (H&E) staining. There were 242 megakaryocytes in the whole film (2.5 cm × 2.0 cm). Classification: Immature megakaryocytes 2/50, granular megakaryocytes 39/50, plate-producing megakaryocytes 9/50, and scattered platelets are rare. Diagnosis: The bone marrow results were consistent with thrombocytopenia.

On day 16, the patient's thyroid-stimulating hormone showed decreased levels of T3 (0.47 nmol/L, normal range: 1.4–3.7 nmol/L), free T3 (2.54 pmol/L, normal range: 5.1–10.1 pmol/L) and TSH (0.017 mIU/L, normal range: 0.64–6.27 mIU/L), and she was positive for anti-TG antibodies (TGAb) and anti-thyroid-peroxidase antibodies. Ultrasound examination showed a weak-echo nodule in the left lobe of her thyroid gland, which led to a suspected diagnosis of asymptomatic Hashimoto thyroiditis (Fig. 3). Therefore, levothyroxine was added as a treatment. Levothyroxine therapy was started on day 3 (on the 18th day of treatment). The platelet count increased to 133 × 10^9^/L (Fig. 2), and mucocutaneous bleeding went into remission. The results of bone marrow examination also suggested a diagnosis of immune thrombocytopenia (Fig. 4).

During the next 2 months follow-up, the thyroxine hormone levels and platelet counts were maintained at normal levels. The child continued to receive levothyroxine, but the prednisone acetate was gradually reduced to once every other day. We discontinued levothyroxine and prednisone in subsequent follow-up visits. Whole-exome sequencing and SLE-related screening were performed in the patient and her father, and the results were normal. Serological testing results for anti-DNA, ACA-IgG, ACA-IgA, ACA-IgM, anti-C1q IgG, anti-β2GPI, anti-AnuA, anti-dsDNA, ANA, AHA, anti-ENA, anti-U1RNP, anti-SSA, anti-SSB, anti-Scl-70, anti-J0-1, and anti-ribosomal phosphoprotein antibodies were negative.

## 3 Discussion

ITP is one of most commonly acquired bleeding diseases in children, and it is currently defined as an acquired autoimmune disease that causes isolated thrombocytopenia.^[1]^ Secondary ITP is caused by medications or concurrent diseases, such as infections, autoimmune conditions, and lymphoproliferative diseases.^[8]^ The responses to therapy for secondary ITP differ from primary ITP because of the diverse causes. Therefore, accurate diagnosis is essential. Corticosteroids, IVIG or anti-D immunoglobulin are the recommended first-line treatment for children, and thrombopoietin receptor agonist treatment, rather than rituximab or splenectomy, is recommended if they have a poor response.^[9]^

Our patient responded poorly to first-line ITP treatment during the emergency observation period, and eltrombopag was added. Eltrombopag is a thrombopoietin receptor agonist that has been frequently used for the treatment of chronic ITP for over a decade but it is rarely used in patients with newly diagnosed ITP.^[10–13]^ The outcomes of eltrombopag treatment as the first-line treatment for children with newly diagnosed ITP were not reported, except for one patient.^[14]^ We first described in detail the use of eltrombopag in a newly diagnosed steroid and IVIG nonresponsive pediatric ITP patient. We used 25 mg as the starting and maintenance dose. The safety and efficacy of eltrombopag are widely reported. The most common side effects of eltrombopag treatment in children are elevated liver enzymes, headache, upper respiratory tract infection, and diarrhea.^[15,16]^ No adverse reaction was observed in our patient.

The symptoms were not controlled by the first-line and second-line therapies in our patient during the initial stage of treatment. ITP in children is frequently triggered by infections or autoimmune diseases, and the underlying causes of ITP were investigated.^[17]^ Blood examinations confirmed that the patient had *M. pneumoniae* infection and likely had *H. pylori* and HSV infection. ANA was also positive with a titer of 1:100. *H. pylori* infection associated with ITP was identified previously.^[18]^ However, ITP secondary to *M. pneumoniae* infection was rarely reported, and primary HSV infection was reported in only 2 reports published decades ago.^[19–26]^ Patients with ITP coexisting with this infection often have refractory disease, and some of them have a poor prognosis.^[19–24]^

However, whether the eradication of infections in patients with ITP effectively increases platelet counts is controversial.^[27]^ Because the persistent thrombocytopenia may be the consequence of infections, we added azithromycin, valaciclovir, amoxicillin, clarithromycin, and PPI therapy. Unfortunately, the platelet counts did not increase, which is inconsistent with other reports. Many studies found ANA positivity in patients with ITP, especially adult patients.^[28,29]^ However, ANA positivity is not sufficient to identify ITP patients who are at risk of developing autoimmune diseases and have a poor response to therapy.^[29]^ Our patient initially exhibited a positive ANA test, but it was negative at the follow-up visits.

The coexistence of thyroid dysfunction (primarily hyperthyroidism) and ITP was well documented in recent years, especially in adult chronic ITP patients, but the pathogenesis is not well understood.^[6,30,31]^ Positive antithyroid antibodies in ITP patients were documented in reports and studies, but Hashimoto thyroiditis as the cause of newly diagnosed ITP is a very rare phenomenon.^[31]^ There were no reports of children with newly diagnosed ITP in combination with Hashimoto thyroiditis.^[32]^ Giordano et al. performed a multicenter retrospective study and showed antithyroid antibodies in 11.6% of children with chronic ITP.^[30]^ Anti-thyroid antibody positivity was also a prognostic factor for the chronicity of ITP, but only 1 of 86 patients needed levothyroxine therapy in the follow-up.^[30]^ Wu et al reported that 2.65% of Taiwanese ITP patients had hypothyroidism, but they did not obtain details of medical conditions of thyroid diseases.^[33]^

Whether the treatment of thyroid disease affects the clinical outcome of ITP is debatable. Many reports show a good response to first- or second-line ITP treatment after thyroid disease resolution and restoration of the euthyroid state.^[1,34]^ Tahir et al reported that several months of steroid therapy failed in a patient who was diagnosed with ITP and coexisting Hashimoto thyroiditis.^[31]^ The platelet count significantly improved with the start of levothyroxine treatment.^[31]^ A recent clinical retrospective study in Japan also confirmed this finding.^[6]^ The platelet count was 7 × 10^9^/L on the 16th day in our patient, and further examinations, including thyroid-stimulating hormone and bone marrow examination, were performed. Our patient had positive TPOAb and TGAb and an abnormal thyroid ultrasound, and she was diagnosed with Hashimoto thyroiditis without any clinical symptoms of hypothyroidism. We added levothyroxine therapy, and the platelet count rapidly returned to normal on the 18th day and remained normal the following day. Our results also found that treatment of the underlying thyroid disorders improved the increase in platelet count in patients with newly diagnosed ITP.

The cause of Hashimoto thyroiditis is genetic susceptibility and environmental risk factors.^[35]^ However, the impact of viral and bacterial infections on Hashimoto thyroiditis is controversial. Several studies indicated that HSV and *H. pylori* infection enhanced the risk for Hashimoto thyroiditis.^[36,37]^ These infections may trigger the production of autoantibodies, which often destroy multiple organs.

Why a significant portion of ITP patients are refractory to first- or second-line treatments is not known. Unfortunately, no reliable factors to help predict the failure of routine treatments in ITP were identified. Further understanding of the cause of the disease in individual patients may help guide treatment.

## Acknowledgments

We thank AJE (https://secure.aje.com/cn/) for polishing the language of this manuscript. (Order Number 1K7MT8XT).

## Author contributions

**Conceptualization:** Zhiqing Tian, Dongqiong Xiao, Xihong Li.

**Funding acquisition:** Zhiqing Tian, Hu Gao, Dongqiong Xiao, Xihong Li.

**Investigation:** Zhiqing Tian, Hu Gao, Dongqiong Xiao, Xihong Li.

**Methodology:** Zhiqing Tian.

**Project administration:** Xihong Li.

**Resources:** Zhiqing Tian, Hu Gao, Dongqiong Xiao, Xihong Li.

**Software:** Zhiqing Tian, Hu Gao, Dongqiong Xiao, Xihong Li.

**Supervision:** Zhiqing Tian, Dongqiong Xiao, Xihong Li.

**Validation:** Zhiqing Tian, Hu Gao, Dongqiong Xiao, Xihong Li.

**Visualization:** Zhiqing Tian, Dongqiong Xiao, Xihong Li.

**Writing – original draft:** Zhiqing Tian, Hu Gao, Dongqiong Xiao, Xihong Li.

**Writing – review & editing:** Zhiqing Tian, Xihong Li.
